# A Note upon Carbo Vegetabilis

**Published:** 1889-02

**Authors:** 


### A NOTE UPON CARBO VEGETABILIS.

Carbo vegetabilis is one of those grand remedies which owes its development to the genius of Hahnemann. It is not a little curious to note how valuable and how deeply acting are many of these Hahnemannian remedies. Calcarea, Silicea, Graphites, etc., are a few of these grand remedies, without which Homœopathy would be much weaker, and many diseases, now curable, would be beyond our curing. Let us then be duly thankful to Hahnemann for his wonderful discovery of drug potentiation.

Carbo vegetabilis is most frequently called for in diseases of those persons who are of a scrofulous or bilious constitution, and who are "run down," as the phrase is, or whose vital powers are weakened by disease or from excesses. This debility may be of the kind known as the "typhoid" condition or go so far as to be called "collapse;" in both of these conditions Carbo vegetabilis is one of the remedies to be studied. In this collapsed condition the patient will be found to be cold, especially the extremities, also the face, nose, ears; even the breath is cold to the hand, the skin is blue, lips also, the pulse weak and intermittent; in some cases the patient may want to be fanned. This condition, occurring after a long illness or after severe hemorrhage, is apt to be best met by Carbo vegetabilis; but China must not be overlooked. If the patient has been previously drugged by Quinine, then, of course, we should use Carbo-veg. for this collapse, otherwise the choice between these two drugs must be decided by the other symptoms. With China the debility is said to be of functional origin; with Carbo-veg. it is of organic origin. Both remedies have hemorrhages and both have them from almost any orifice of the body; with China the flow is more active and is apt to be clotted; with

Carbo-veg. the flow is slow and passive and more liquid than that of China.

The Carbo-veg. patient has been very sick ; he is run down, is very weak from his disease, his blood and tissues are about disorganized, hence the tissues of the parts from which the discharge comes, whether blood or pus, are diseased. The patient is, therefore, weak both from his low condition and from the discharges. The China patient, on the other hand, is not so low or weak previous to the occurrence of the discharge ; he is weakened by the excessive loss of the vital fluids, which is apt to have suddenly occurred.

In chronic cases, where a cure is impossible, these two remedies are often able to bring some relief to the sufferer. Two instances of this may be appropriately related here. A thin, lean man of about sixty years had suffered from heart disease, caused most probably by constantly lifting heavy boxes ; he could not lie down, had to spend his days and nights sitting up in a chair. Large, flat ulcers formed on his swollen legs, which constantly discharged clear water. Occasional attacks of very severe dyspnœa would occur, when patient would seem to be fairly gasping for his last breath, the skin would get cold and clammy, nose and ears very cold, yet he would constantly demand to be fanned and to be fanned very rapidly. Carbo-veg. would relieve these attacks promptly and sometimes to such a degree that the poor fellow could lie down for awhile—a most welcome respite to a man condemned to spend months sitting in a chair.

The other case was in a young man, far gone with phthisis. We had been traveling and were delayed over twenty-four hours. During this time this consumptive had not had one good meal; to make up for this he made quite free potations from a bottle of whisky. At the end of this twenty-four hours we stopped at a hotel for supper and to await the next train. At the table this patient ate some pickles, which made him sick and he vomited all the supper he had just taken. About a half-hour after this vomiting I was asked to see the patient, as he was thought to be dying. I found him wrapped up in his overcoat sitting before a blazing wood fire, his face cold, chin and ears ditto, lower jaw dropped, breathing rather heavily. He said he was dying and wanted me to send messages, etc., to his family. In two hours our train was due. He wanted to travel on it if possible and so did I. One dose of China$^{200}$, was given that man. The result was that he *did* travel on that train and moreover he traveled all that night and until twelve o'clock the next day without eating or having any proper sleep, as the train was largely overcrowded and accommodations could not be obtained. When I again saw that man, twenty-four hours later, he was as lively as a cricket.

A prominent symptom accompanying the discharges of Carbo-veg. is a burning pain. We find this burning across the sacral region in women who have hemorrhages from the uterus; we find it in the ulcers; the stool is burning; in the chest we find it even more markedly, being described as "burning as from glowing coals;" we also read of burning in the "stomach, spreading down to small of back and up to the shoulders;" in the abdomen it is "burning, lancinating in epigastrium and deep in abdomen." This burning reminds us of Arsenicum, which has it so markedly; Arsenic has more restlessness than Carbo-veg.; the Arsenic symptoms resemble those of Carbo-veg. at many points, as in the hemorrhages, in gastric ailments, in diarrhœa, etc. The general distinction between the two drugs is in the restlessness, the irritability, the anxiety, the thirst, etc., of the Arsenic patient.

The discharges, the diarrhœa, etc., of this drug are apt to be fetid; ulcers which are flat, not deep, with a mottled skin around them, discharging a thin, ichorous, burning fluid; these ulcers burn so at night that the patient cannot sleep.

When we find loose, rattling rales in the chest of debilitated persons, accompanied by signs of that cold, weak, collapsed condition just mentioned, then Carbo-veg. is probably the remedy; also in the "asthma" of old or otherwise debilitated persons. They are weak and trembling; look as if they were dying; maybe they are cold in legs, ears, etc., yet want to be fanned; maybe the abdomen is packed full of gas, and they cannot belch it up. Antimonium-tart. also has this loud rattling of mucus in the chest, which seems to be full of mucus, yet little or none is expectorated. The patient may finally show signs of cyanosis from this mucus filling up the air-passages; the patient gets cold and blue and maybe drowsy. This is a case for Antimonium-tart.

In dyspeptic ailments, Carbo-veg. is very frequently called for, especially when they are due to indulgence in rich food or in wines, etc. There is great flatulency, constant passing of flatus, frequent eructations, which relieve; has protruding, blue hemorrhoids which burn. In these cases this remedy is apt to be indicated after Nux vomica. With rheumatic pains in the limbs we find this flatulency.

Carbo vegetabilis symptoms are generally better from cold and worse from heat; Carbo animalis has just the reverse. Carbo-veg. acts chiefly upon the upper right and lower left sides; Carbo-an. acts chiefly upon the upper left and lower right sides.

